# Rural chronic disease research patterns in the United Kingdom, United States, Canada, Australia and New Zealand: a systematic integrative review

**DOI:** 10.1186/s12889-020-08912-1

**Published:** 2020-05-24

**Authors:** R. Disler, K. Glenister, J. Wright

**Affiliations:** Department of Rural Health, Faculty of Medicine, Dentistry and Health Sciences, University of Melbourne, ‘The Chalet’ Docker street, Wangaratta, VIC 3677 Australia

**Keywords:** Chronic disease, Rural and remote, Rural healthcare, Referral, Patterns of referral

## Abstract

**Background:**

People living in rural and remote communities commonly experience significant health disadvantages. Geographical barriers and reduced specialist and generalist services impact access to care when compared with metropolitan context. Innovative models of care have been developed for people living with chronic diseases in rural areas with the goal of overcoming these inequities. The aim of this paper was to describe the characteristics and outcomes of studies investigating innovative models of care for people living with chronic disease in rural areas of developed countries where a metropolitan comparator was included.

**Methods:**

An integrative systematic review was undertaken. *P*referred *R*eporting *I*tems for *S*ystematic Reviews and *M*eta-*A*nalyses (PRISMA) method was used to understand the empirical and theoretical data on clinical outcomes for people living with chronic disease in rural compared with metropolitan contexts and their models of care in Australia, New Zealand, United States, Canada and the United Kingdom.

**Results:**

Literature searching revealed 620 articles published in English between 1st January 2000 and 31st March 2019. One hundred sixty were included in the review including 68 from the United States, 59 from Australia and New Zealand (5), 21 from Canada and 11 from the United Kingdom and Ireland. 53% (84) focused on cardiovascular disease; 27% (43) diabetes mellitus; 8% (12) chronic obstructive pulmonary disease; and 13% (27) chronic kidney disease. Mortality was only reported in 10% (16) of studies and only 18% (29) reported data on Indigenous populations.

**Conclusions:**

This integrated review reveals that the published literature on common chronic health issues pertaining to rural and remote populations is largely descriptive. Only a small number of publications focus on mortality and comparative health outcomes from health care models in both urban and non-urban populations. Innovative service models and telehealth are together well represented in the published literature but data on health outcomes is relatively sparse. There is significant scope for further directly comparative studies detailing the effect of service delivery models on the health outcomes of urban and rural populations. We believe that such data would further knowledge in this field and help to break the deadly synergy between increased rurality and poorer outcomes for people with chronic disease.

## Background

Chronic disease prevalence and outcomes differ between urban and metro populations in Westernised countries, despite the presence of developed world health systems in urban and rural regions. In the United States (US), rural populations have higher levels of chronic disease compared to urban dwellers [1]; in Australia, poor health outcomes and mortality rates are higher for populations in rural areas compared to metropolitan areas, with the worst outcomes for those living in remote areas [2]. Other geographical trends in health outcomes can be observed in developed countries such as the increased incidence in premature death in rural counties of Southeast and Southwest US [3].

Common chronic health conditions such as cardiovascular disease (CVS), diabetes (DM), chronic obstructive pulmonary disease (COPD) and chronic kidney disease (CKD) are known to illustrate these disparities. In the US, a significantly higher prevalence of congestive cardiac failure was observed among general practice patient populations in rural areas than in both capital cities and metropolitan areas [4]. Increased admission rates for congestive cardiac failure in rural areas compared to metropolitan areas has also been demonstrated in Australia [5]. Furthermore, geographical variations in CVS disease prevalence have also been observed in the United Kingdom (UK) [6].

Diabetes management in the rural US, was shown to be less likely to reach blood pressure or HbA1c targets, and renal and ophthalmic screening requirements when compared to urban patients [7]. One Australian study also demonstrated a doubling of the incidence of DM in the 15 years to 2005 in rural Victoria [8]. In the US, age-adjusted prevalence of COPD was higher for rural residents, as were rates of hospitalisation and mortality [9]. The higher hospital admissions with COPD in rural areas has also been demonstrated in Australia [10]. Patients with CKD in a Canadian study living 50 km or more from a nephrologist were less likely to be on appropriate medications and more likely to die or be hospitalised that those who lived closer to specialist nephrologists [11]. Remoteness was also found to be associated with increasing mortality among patients initiating chronic haemodialysis treatment in the US [12].

From such studies as those detailed above, and many more like them, across a range of chronic health conditions and across a range of developed countries, significant differences in disease prevalence and outcomes are present between urban and rural populations. Certain factors are intuitively part of the association with these inequities. These include distance from health services, and reduced access to both primary and specialist care clinicians. Additional factors which may be classed as social determinants of health include reduced socio-economic status and reduced health literacy in many rural populations.

Across developed countries, definitions of urban, regional, rural and remote geographical areas vary. Nomenclature for what constitutes a rural or urban area often does not consider important cultural or economic differences across geographical areas which require a detailed understanding for the formation of effective health policy formation [13]. Studies detailing differences in urban and rural health outcomes often simply recognise different population subsets based on geography and population density.

Given such disparities in chronic health between urban and non-urban populations one would anticipate that published research would enhance our understanding of this phenomena and detail studies which lead to a realisation of why such disparities occur despite the panoply of treatments available for chronic health conditions. Of particular interest would be published studies of health care initiatives which have both metropolitan and regional/remote arms so that outcomes for the same intervention can be directly compared.

This paper details the current state of knowledge of chronic health conditions across several developed countries, all of which have recognised disparate health outcomes between urban and non-urban populations. We believe a thorough review of current knowledge is required to plan further work to reduce disparities in health care outcomes for common chronic health conditions in non-urban populations. This integrated review aimed to review and amalgamate published empirical and theoretical data, inclusive of qualitative and quantitative methodologies, on clinical outcomes and models of care of people living with CVD, DM, COPD and CKD in regional and rural compared with metropolitan contexts in Australia, New Zealand, Canada, US and the UK. This paper aims to identify knowledge gaps in our understanding of health disparities and how further research in this area may benefit regional and remote populations.

## Methods

### Design

Systematic approach following the *P*referred *R*eporting *I*tems for *S*ystematic Reviews and *M*eta-*A*nalyses (PRISMA) method. We considered an integrative review the most appropriate review for this research question, in order to consider both qualitative and quantitative data and to assess knowledge in the field in a systematic manner.

### Eligibility criteria

Papers were included if they were:

- published in peer reviewed journals between January 2000 and 31 March 2019;
- written in the English language;
- reported management of chronic disease within the rural context in Australia, New Zealand, Canada, US or the UK. These countries were chosen due to their similarities in organisation of health system and similarities in geographical distribution of densely populated metropolitan centres and sparsely populated rural populations.

Papers written prior to January 2000 were excluded to ensure consistency with current clinical approaches. Original research papers, systematic reviews, and reviews were considered. Opinion pieces and case studies were excluded from this review. Papers were excluded if data was published in a way that rural data could not be deciphered from overall reported data. Papers were included if they reported data on rural health care, rural in this context was accepted if it met the definitions noted above and/or was defined by the authors as reporting on rural and remote contexts relative to country of origin.

### Information sources

**Fig. 1:**
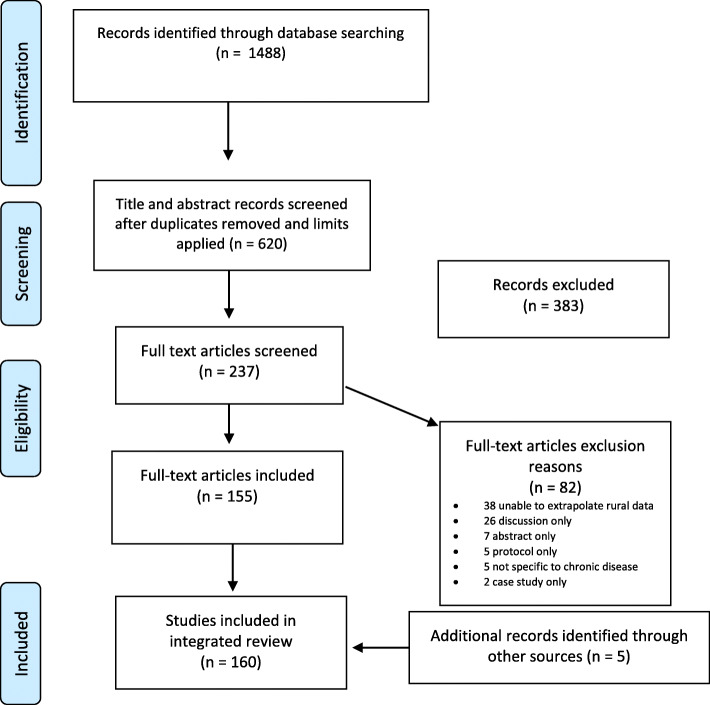
PRISMA Flow Diagram. *From:* Moher D, Liberati A, Tetzlaff J, Altman DG, The PRISMA Group (2009). *P*referred *R*eporting *I*tems for *S*ystematic Reviews and *M*eta-*A*nalyses: The PRISMA Statement. PLoS Med 6 (6): e1000097. doi:10.1371/journal.pmed1000097

The electronic databases MEDLINE, CINHAL, Embase, Informit and Google Scholar were searched using Medical Subject Headings (MeSH) and key words. Figure 1 outlines an example Medline Ovid search strategy; this was translated into all other databases. The search terms and strategy were reviewed by a health informatics expert with the following terms and appropriate derivatives were used: *‘rural health’ AND ‘chronic disease’.* Separate searches were completed for the four included chronic conditions, with derivatives used for: ‘*cardiovascular disease*’, ‘*COPD’*, ‘*diabetes mellitus’, ‘hypertension’* and ‘*renal disease’*. The World Wide Web was searched using Google Scholar and Google search engine for related electronic documents.

### Study selection

Papers were assessed for eligibility for inclusion by two independent investigators (RD and JW) with consensus gained from a third independent investigator as necessary (KG).

### Data extraction

Data were extracted by single investigators using a structured data extraction Table (RD) and accuracy of data checked by an independent investigator (JW). The data extraction table included: author details; journal details; methodological approach; location, service and population size; data utilised and data sources; summary of findings; and conclusions.

## Results

### Characteristics of included studies

Literature searching revealed 1488 papers. After duplicate removal 620 remained that were published in English between 1st January 2000 and 31st March 2019 (Fig. 1). Three hundred eighty-three were excluded on record title and abstract review and a further 82 were excluded after full text review: 38 were unable to extrapolate rural data; 26 discussion only; 7 presented only preliminary abstract data; five were study protocols; five did not specify specific chronic diseases; and two further presented case studies. Five further papers were included from hand searching of article reference lists and key journals. A total of 160 papers were included in the review with one (1%) systematic reviews; five (4%) randomised controlled/cluster trials, 141 (88%) observational descriptive studies, 12 (8%) qualitative studies, and one mixed methods (Table 1).

**Table 1:** Characteristics of included studies

CHARACTERISTIC	f	%
**Region**		
Australia and New Zealand	59	37
Canada	21	13
UK and Ireland	11	7
US	68	43
Comparative data from Australia and Canada	1	1
*n* = 160		
Design	**f**	**%**
Systematic review	1	1
Randomised controlled/cluster trial	5	4
Observational descriptive	140	88
Qualitative	12	8
Mixed methods	1	1
Discussion	1	1
*n* = 160		
**Condition**	**f**	**%**
CVD	84	53
COPD	12	8
DM	43	27
CKD	21	13
*n* = 160		
**Reporting of mortality**		**%**
No	144	90
yes	16	10
*n* = 160		
**Reporting of Indigenous data**^**a**^		**%**
No	120	81
Yes	29	19
*n* = 149		
**Reporting of service models**		**%**
No	112	70
Yes	48	30
*n* = 160		
**Reporting of Telehealth**		**%**
No	144	90
Yes	16	10
*n* = 160		

^**a**^**Countries include: Australia, Canada, New Zealand, US**

**Fig. 2:**
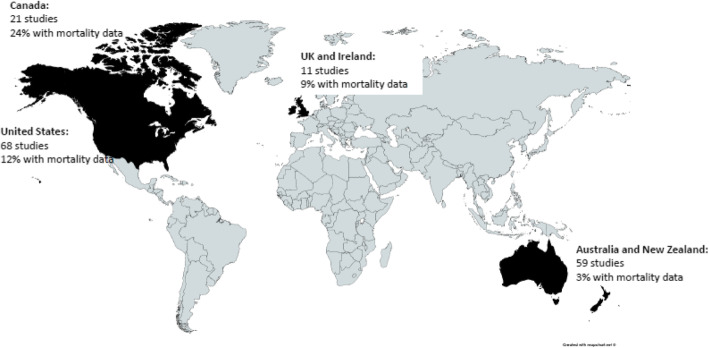
Geographical representation (Figure created using MapChart.net)

Sixty-eight of the included papers originated from the US, 59 from Australia and New Zealand [4], 21 from Canada and 11 from the UK and Ireland. A single study also compared Australian and Canadian data. In regards to the health condition studied, over half focused on CVD (53%, 84); a quarter on DM (27%, 43), with COPD (8%, 12) and CKD (13%, 27) also represented (Fig. 2).

### Mortality as an outcome

Only 10% [14] of the included papers that measured mortality within their outcomes. Canada had the highest proportion of papers reporting mortality outcomes (5 of 16, 24%). The US and UK and Ireland reported mortality in 12 and 9% of their papers respectively, with Australia and New Zealand reporting mortality in only 3% of their rural papers on chronic disease.

### Reporting of indigenous outcomes

Outcomes relating to Indigenous populations were scarce across the papers from the US, Canada, Australia, and New Zealand, with only 29 of 149 (19%) papers including data in this context, and with 8 of these papers only representing this population within baseline characteristics without extrapolating differences in outcomes. Australia and New Zealand had the highest proportion of Indigenous reporting, with 21 of 59 (14%) papers including Indigenous data.

### Reporting of service models in the rural context

Innovative service models are of increasing interest in the search for effective and sustainable approaches in rural healthcare with 48 (30%) of papers reporting on various models of care. Telehealth care is similarly of increasing interest in addressing access to care and streamlining assessment in remote locations, with 16 (10%) papers found in this review directly addressed the use of telehealth in the rural context.

## Discussion

This integrated review revealed that the literature on common chronic health issues pertaining to rural and remote populations was largely descriptive. Only a small number focused on mortality and comparative health outcomes between urban and non-urban populations, and similarly few discussed health care models that could be equally applicable across these contexts. Innovative service models and the use of telehealth were well represented, but again data on health outcomes from such models was relatively sparse.

The disparities in chronic health outcomes in developed countries is a significant issue which is universally acknowledged by governments and health services. The desire to provide an adequate health service for all and particularly equity of access to health care for all populations is accompanied by geographical practicalities and maldistribution of workforce that make such provision difficult to realise. Inequities in Indigenous health outcomes are also influenced by these issues. Population statistics demonstrate the disparities in terms of prevalence of, and outcomes of chronic health conditions. However, these statistics do little to explain why these disparities occur and how progress may be made to reduce them. Furthermore, national trends in improving chronic health disease outcomes such as the 40% reduction in mortality from coronary heart disease seen in the US from 1999 to 2009 conceal persisting disparities for rural areas [15]. Obesity, a factor underlying many chronic health conditions is more common among adults in rural areas compared to those in urban areas [16]. Yet studies which have controlled for obesity and other risk factors such as poverty and tobacco use still show a higher prevalence of CVD in rural areas [17].

Several studies included in this review included rural populations in addition to urban population outcomes but did not present comparative data beyond baseline prevalence. It would be ideal for chronic health studies to represent both urban and rural populations so that direct outcome comparisons can be drawn from these two populations. In future studies it may be possible to build in metropolitan comparators to the study design. Perhaps health care outcomes from telehealth initiatives in remote areas could be compared to usual care in metropolitan centres to see if equivalent outcomes were achievable from emerging telehealth initiatives.

Differing systems of health service funding through taxation and different models of public and private care across various countries is also highly likely to have bearing on the data presented. Indeed, the socio-economic cost of some service delivery models was a major feature in this review. The Centre for Disease Control and Prevention, for example, identified having health insurance as a leading health indicator of health outcomes in the US [14], yet people living in non-metropolitan areas are more likely to be uninsured that their metropolitan counterparts [18]. In Australia there is an inequity of bulk billed (no cost to patients, with fixed national healthcare rebate paid to practice) general practices in rural and remote areas [19]. Patient access to medical services based on different health service funding models is a major factor in determining health outcomes.

Inequalities of access to health care services along the rural-urban continuum may be masked in large scale evaluations [20]. Distance from health services is one factor important in determining access, but service delivery models and funding models are also important and well represented in the published literature interrogated for this study. Additionally, the countries represented in this review all had different classifications of urban, regional and remote based on their own unique geography. Such taxonomies simplify the complex interplay of factors which may play a part in urban-rural heath disparities [13]. It is possible that the classifications of urban, regional or remote, may have been applied differently across the publications, which may impact on the interpretation and reporting.

A key issue raised was the lack of data specific to Indigenous populations within the included developed countries. While there is clear recognition of the heightened burden of disease within Indigenous populations, and in particular those within rural communities, there was a distinct lack of reporting across the studies reviewed. Clear evidence is provided as to the high burden of chronic disease borne by Indigenous Australians, for example. A study by Azzopardi for example showing that burden of DM for Indigenous children and adolescents is greater that for non-Indigenous people regardless of geographical location, but that young Indigenous people living in remote communities are clearly at excess risk of DM [21]. Despite the recognised issues, only 19% of studies included indigenous data at all, diminishing visibility and opportunity to address chronic disease patterns and issues within these populations.

This integrated review demonstrates that whilst there are many fascinating examples of innovative health care provision for patients in rural and remote areas, there is very little directly applicable comparative data on outcomes of initiatives in metropolitan and non-metropolitan populations. Whether such studies would demonstrate different outcomes is open to speculation but were they to do so, understanding of the effects of remote geography on healthcare would be likely to be enhanced.

There are several limitations of this review. As in all reviews of this nature it is possible that our search has not identified all relevant literature. However, we believe the systematic prospective review process, including the use of broad search terms and screening and data extraction by two independent researchers, provided a rigorous approach to capture papers that included relevant information. While selecting developed countries with similar health systems provided for more appropriate comparisons, this also limits the scope for generalisation beyond the identified regions. The heterogeneity of the included studies and contexts has also recognised as a challenge, however this paper does provide some insight and documentation of what data is being published and the gaps that currently are faced.

## Conclusion

In conclusion, this review demonstrates that there is significant scope for further directly comparative studies detailing the effect of service delivery models on the health outcomes of urban and rural populations. We believe that such data would further knowledge in this field and help to break the deadly synergy between increased rurality and poorer outcomes for people with common chronic health conditions.

## Supplementary information

**Additional file 1.** Characteristics of included studies.

## Data Availability

All data generated or analysed during this study are included in this published article [and its supplementary information files].

## Abbreviations

CKD: Chronic kidney disease
COPD: Chronic obstructive pulmonary disease
CVS: Cardiovascular disease
DM: Diabetes Mellitus
MeSH: Medical subject headings
PRISMA: *P*referred *R*eporting *I*tems for *S*ystematic Reviews and *M*eta-*A*nalyses
UK: United Kingdom
US: United States
